# Evolution of RNA viruses from SARS to SARS-CoV-2 and diagnostic techniques for COVID-19: a review

**DOI:** 10.1186/s43088-021-00150-7

**Published:** 2021-10-06

**Authors:** Johra Khan, Lubna Ibrahim Al Asoom, Maryam Khan, Ishani Chakrabartty, Sayequa Dandoti, Mithun Rudrapal, James H. Zothantluanga

**Affiliations:** 1 Department of Medical Laboratory Sciences, College of Applied Medical Sciences, Majmaah University, Majmaah, 11952 Saudi Arabia; 2 Physiology Department, College of Medicine, Imam Abdulrahman Bin Faisal University, Dammam, 31541 Saudi Arabia; 3 Department of Biochemistry, Faculty of Life Sciences, Aligarh Muslim University, Aligarh, Uttar Pradesh 202002 India; 4 Department of Science, P.A. First Grade College (Affiliated To Mangalore University, Mangalore), Nadupadav, Mangalore, Karnataka 574153 India; 5 Department of Biology, Deanship of Preparatory Year, Imam Abdulrahman Bin Faisal University, Dammam, 31541 Saudi Arabia; 6 Department of Pharmaceutical Chemistry, Rasiklal M. Dhariwal Institute of Pharmaceutical Education & Research (Affiliated to Savitribai Phule Pune University, Pune) , Chinchwad, Pune, Maharashtra 411019 India; 7 Department of Pharmaceutical Sciences, Faculty of Science and Engineering, Dibrugarh University, Dibrugarh, Assam 786004 India

**Keywords:** ssRNA viruses, SARS, MERS, SARS-CoV-2, COVID-19, Diagnostics

## Abstract

**Background:**

From the start of the twenty-first century up to the year 2021, RNA viruses are the main causative agents of the majority of the disease outbreaks the world has confronted. Recently published reviews on SARS-CoV-2 have mainly focused on its structure, development of the outbreak, relevant precautions, management trials and available therapies. However, in this review, we aim to explore the history, evolution of all coronaviruses and the associated viral outbreaks along with the diagnostics for COVID-19 in the twenty-first century.

**Main body:**

We have focused on different RNA viruses’ viz. SARS-CoV, MERS-CoV, and SARS-CoV-2, their classification, and the various disease outbreaks caused by them. In the subsequent section, the comparison of different RNA viruses affecting humans has been made based on the viral genome, structure, time of the outbreak, mode of spread, virulence, causative agents, and transmission. Due to the current mayhem caused by the rapidly emerging virus, special attention is given to SARS-CoV-2, its genome updates, and infectivity. Finally, the current diagnostic techniques such as nucleic acid testing (real time-polymerase chain reaction and loop-mediated isothermal amplification), CRISPR-based diagnostics (CRISPR based DETECTR assay, CRISPR based SHERLOCK test, AIOD-CRISPR, FELUDA, CREST), chest radiographs (computed tomography, X-ray), and serological tests (Lateral flow assay, enzyme-linked immunosorbent assay, chemiluminescent immunoassay, neutralization assay, nano-sensors, blood test, viral sequencing) with their pros and cons, and future diagnostic prospective have been described.

**Conclusions:**

The present gloomy scenario mandates clinical manifestations, contact tracing, and laboratory tests as important parameters that need to be taken into consideration to make the final diagnosis.

## Background

Coronaviridae comprises a family of around 40 coronaviruses wherein most of the members of this family are known to cause diseases in animals [1]. At the beginning of the twenty-first century (in the year 2003), a severe acute respiratory syndrome (SARS-CoV) outbreak was reported in China and Hong Kong [2]. In 2012, the second outbreak, known as the Middle East respiratory syndrome (MERS-CoV) occurred in the regions of the Middle East and the Republic of Korea [3]. The chain of the outbreak has continued, and in December 2019, the world witnessed the COVID-19 pandemic caused by a novel coronavirus Severe acute respiratory syndrome coronavirus 2 (SARS-CoV-2), with a high mortality rate. These RNA viruses are highly infectious, owing to the high rate of mutation and short generation time that leads to their rapid evolution. The origin of RNA viruses is indistinct. Many pieces of evidence show that the RNA viruses had evolved from few DNA viruses and developed within some of their vertebrate hosts over millions of years ago [4]. When the evolution of RNA viruses was traced based on gene sequences and rate of evolutionary changes (nucleotide substitution), it was concluded that the families of RNA viruses that are found today might have appeared very recently, probably not more than 50,000 years [5].

Recently published reviews on the emerging SARS-CoV-2 have mainly focused on its structure, development of the outbreak, relevant precautions, management trials and available therapies [6]. However, in this review, we aim to explore the history and evolution of all coronaviruses and the associated viral outbreaks in the twenty-first century. We have also critically compared the pathogenesis and epidemiology of the targeted coronaviruses, with a special focus on the diagnostic techniques used for the detection of various RNA viruses.

## Main text

### RNA viruses and their classification

RNA viruses are broadly classified as positive-sense RNA (ssRNA+) viruses, negative-sense RNA (ssRNA−) viruses, and a third class that co-exists and comprises both positive and negative sense molecules, particularly known as arenaviruses [7]. The replication in RNA viruses takes place by the generation of messenger RNA (mRNA) from their genome. The mRNA synthesizes numerous polyproteins that are cleaved into multiple proteins using either viral or cellular protease enzymes. These viruses have the genetic codes for the synthesis of an RNA-dependent RNA polymerase [8]. This enzyme then transcribes the +ve RNA strand as well as the complementary −ve RNA strands, which occur as intermediate products of genome replication. During this process, new genomic RNA molecules are produced from the second transcription step [9]. Viruses that contain a continuous, single-stranded, −ve-sense RNA genome must be replicated to produce +ssRNA genome for the synthesis of protein and other viral materials. This is indicative of the fact that the −ssRNA genome is non-infectious. Some retroviruses (HIV) follow reverse transcription to produce dsDNA to translocate into the host nucleus, integrate with its genome, and start replication to produce RNA [10].

**Fig. 1:**
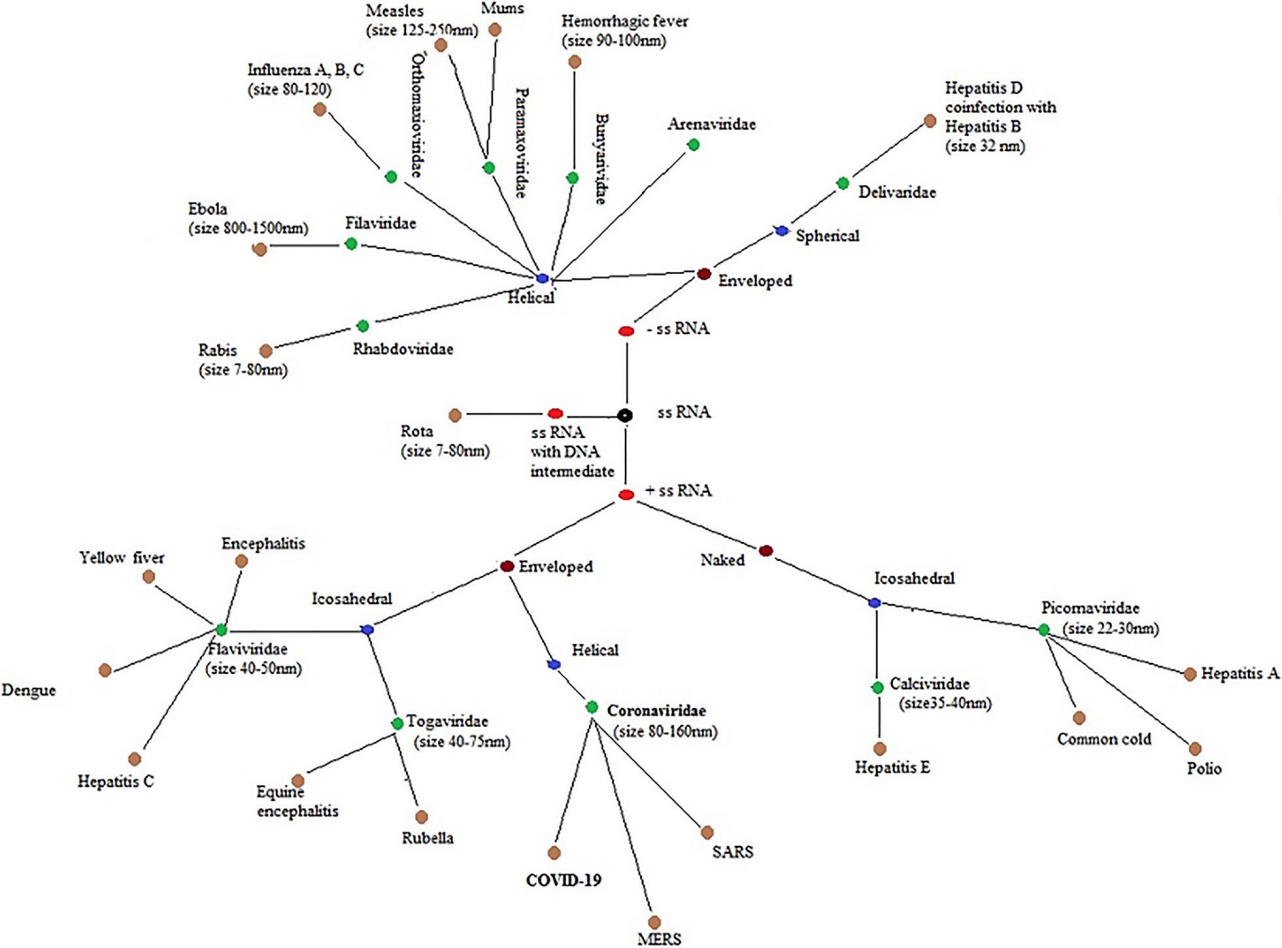
Classification of RNA viruses is shown with different colors. ssRNA is classified mainly to +ssRNA and −ssRNA. Each branch includes multiple viral subtypes that cause different diseases [12]

The classification of RNA viruses into different families depends upon the number, size, position of viral genes in the RNA molecule, the number of polyproteins synthesized at the time of viral infection, and the presence of an envelope as a virion component. Figure 1 illustrates the classification of RNA viruses including their subtypes. Some families of positive sense RNA virus are Picornaviridae, Astroviridae, Caliciviridae, Hepeviridae, Flaviviridae, Togaviridae, Arteriviridae and Coronaviridae. On the other hand, the families of negative-sense RNA include Rhabdoviridae, Bornaviridae, Paramyxoviridae, and Filoviridae [11].

### Virology and pathogenesis of coronaviruses

Coronaviruses (CoVs) are the viruses whose genome structure is best known among all RNA viruses. RNA-based viruses like the coronavirus or the flu tend to mutate around 100 times faster than DNA-based viruses although the coronavirus mutates more slowly than influenza viruses [13]. CoVs have been defined as respiratory tract viruses in the samples collected from the individuals who presented symptoms of respiratory tract infection in 1962 [14]. This is a large family of viruses that are common in many different animal species, including camels, cattle, cats, and bats. CoVs are members of the subfamily “*Coronavirinae*” (Family: *Coronaviridae*; Order: *Nidovirales*) that contains four genera alpha-CoV, beta-CoV, gamma-CoV, and delta-CoV [15]. Gamma and delta CoVs generally infect birds, although some of them can cause infection in mammals. On the other hand, alpha and beta CoVs are known to harm humans and animals. The SARS-CoV (beta coronavirus), 229E (alpha coronavirus), HKU1 (beta coronavirus), NL63 (alpha coronavirus), OC43 (beta coronavirus) and MERS-CoV (beta coronavirus) can cause severe illnesses in humans. However, beta-CoVs are the most important group as it contains the highly pathogenic viruses in humans including COVID-19, MERS-CoV, and SARS-CoV [16]. Rarely, animal CoVs can be transmitted to humans and, as a result, the virus may spread among humans during epidemics such as MERS, SARS, and COVID-19 [17].

### Severe acute respiratory syndrome (SARS)

SARS is a respiratory sickness caused by the zoonotic RNA human coronavirus (CoV) group 2b, SARS-CoV [18]. Angiotensin-converting enzyme 2 (ACE2) was identified as its functional receptor [19]. The definitive animal host responsible for the transmission of the virus is not clear, but human transmission is considered to have originally come about through the masked palm civet, with heavy human interaction in the outdoor Chinese markets [20]. The first outbreak was reported from Guangdong Province in China in 2002, with rapid outspread to Hong Kong and afterward to 33 other countries in over five continents. Healthcare workers were disproportionately affected, due to SARS exposures taking place in hospital settings. By the time the outbreak was controlled in 2004, there were about 8000 confirmed cases and around 800 deaths were reported. The severity of the disease was found more in the elderly people, with a rate of mortality greater than 40% in patients over the age of 60 years. Since 2004, no more infections were detected, and the SARS pandemic was declared to be over [21].

### Middle East respiratory syndrome (MERS)

After approximately 10 years of the occurrence of SARS-CoV, another highly pathogenic CoV known as Middle East respiratory syndrome Coronavirus (MERS-CoV) has emerged in Middle East countries. It was caused by a virus that was originally known as human CoV-Erasmus Medical Centre/2012 (HCoV-EMC/2012), but later it was renamed as Middle East respiratory syndrome coronavirus, MERS-CoV [22]. It was initially isolated from a patient who died of severe respiratory illness in Saudi Arabia in 2012 [23, 24]. Dromedary camels, hosts for MERS-CoV, have been involved in direct and indirect transmission to humans, although the exact mode of transmission is still unknown [25]. Bats are likely the main mammalian reservoir [26]. Evidence shows that MERS-CoV can be transmissible to humans via both animals and humans. However, the consecutive epidemic of MERS shows that the pathogen has dispersed to various parts of the world mainly through inter-human transmission. Human-to-human infection is known to occur when secondary infected patients come in close contact with a primary infected individual. These secondary infected patients may include family members, healthcare workers, and individuals who shared the same hospital room or visited the patients [27].

The WHO reports between 2012 and January 2019 showed that there were 2279 laboratory-confirmed cases of MERS, including 806 associated deaths (case-fatality rate = 35.3%) reported globally. From the Middle East, the virus spread to around 27 countries where some cases are found to be chains of human-to-human infections; for instance, in the Republic of Korea, the first Korean patient affected by MERS-CoV was diagnosed on 20 May 2015 after his return from Qatar [28]. Owing to the secondary mode of transmission, 186 Korean citizens had been infected with MERS-CoV in a short period. Phylogenetic analysis also suggests that the MERS-CoV isolate found in the Korean patient is closely related to the Qatar strain. Although a large number of cases were reported from 27 countries, the majority of cases (i.e., 1901) was reported from Saudi Arabia [25].

### Evolution of the novel corona virus

In December 2019, a new coronavirus emerged in Wuhan, mainland, China. The first infected cases reported a recent visit to Wuhan Huanan Seafood Wholesale Market. The initial investigation and analysis revealed the characteristics of coronavirus similar to SARS and MERS. However, the new coronavirus has some distinct features. WHO declared the newly discovered virus as the seventh coronavirus and named it SARS-CoV-2. COVID-19 is the name given to the disease caused by SARS-CoV-2 [29]

COVID-19 infection was presented first with mild acute respiratory distress syndrome and was taken less seriously than SARS and MERS. China CDC reported a mortality rate of 2% [30]. Time passed and the novel virus showed its ferocity through the high infectivity. According to WHO on the 9th of August 2021, more than 210 countries and territories were affected with a total number of confirmed cases reaching 202,296,216 with a total of 4,288,134 reported deaths (https://covid19.who.int/). A numerical representation of the devastating effect of COVID-19 in 30 of the hardest-hit countries has been highlighted in Table 1.

**Table 1:** Data on the number of confirmed cases and death due to the COVID-19 pandemic as of 9th August 2021

Country	Confirmed case	Deaths
United States of America	35,501,444	611,504
India	31,969,954	428,309
Brazil	20,151,779	562,752
Russian Federation	6,447,750	165,650
France	6,135,076	111,102
The United Kingdom	6,042,256	130,320
Turkey	5,871,884	52,089
Argentina	5,012,754	107,302
Colombia	4,834,643	122,277
Spain	4,566,571	81,931
Italy	4,390,684	128,220
Iran	4,119,110	94,015
Germany	3,790,766	91,784
Indonesia	3,666,031	108,571
Mexico	2,964,244	244,248
Poland	2,884,098	75,285
South Africa	2,523,488	74,813
Ukraine	2,259,151	53,100
Peru	2,124,128	196,873
Netherlands	1,883,513	17,869
Iraq	1,704,363	19,203
Czechia	1,674,906	30,363
Philippines	1,649,341	29,122
Chile	1,623,363	36,016
Canada	1,438,219	26,663
Bangladesh	1,353,695	22,897
Malaysia	1,234,852	10,749
Belgium	1,136,726	25,268
Sweden	1,104,538	14,657
Romania	1,084,919	34,319

### The genome of COVID-19

The genetic analysis of the novel coronavirus revealed a single-stranded positive-sense RNA. This RNA virus is relatively large and with a length of 30 Kb. It belongs to *beta coronaviruses* that include Bat-SARS-like (SL)-ZC45, Bat-SL ZXC21, SARS-CoV, and MERS-CoV [31]. The genomic RNA encodes several proteins; some are structural and others are non-structural. The structural proteins include the envelope (E) with 75 amino acids, the membrane (M) with 222 amino acids, the spike (S) with 1273 amino acids, and the nucleocapsid (N) with 419 amino acids [32]. There are 16 non-structural proteins with a wide range of functions. Most of the non-structural proteins are involved in the formation of the replication transcription complex (RTC). The RTC has a double membrane and multiple cleavage proteases. RTC, inside the infected cell, synthesizes sub-genomic RNA (RNA segments). Each RNA segment has a common 5′-leader and 3′-terminal sequence. The process of transcription which occurs for the genomic segments is known as the regulatory sequences (TRS) [33]. TRS is separated by an open reading frame (ORF) which is responsible for the termination of the transcription process. The termination of transcription is mediated by certain proteases such as chymotrypsin-like protease, main protease, papain-like proteases, in addition to a specific unique protease of COVID-19 that is 3′–5′ exoribonuclease [34]. There are 6 ORF in the coronavirus RNA. The first ORF forms two-third of the whole RNA and encodes the non-structural proteins. The other ORFs form the remaining one-third, and they encode the structural and accessory proteins [35].

When comparing the genome sequence of various CoVs, the similarity was found to be more (about 54%) at the segment encoding the non-structural proteins. However, less similarity, of about 43% was found in the structural protein-encoding segment. This might indicate the possibility of more mutations in the structural protein genomic segment to provide better adaptation to different hosts [36].

The overall genome of COVID-19 was found to be 96.5% similar to that of bat CoV, and 75.6% to SARS-CoV [37]. The similarity of the novel virus genome to that of the bat corona genome increases the suspicion that it originates from bats. However, the first case denied any contact with bats and was only detected in bats that became recently available in the seafood market. Therefore, intermediate hosts are most probably involved and need to be identified urgently [38].

### Infectivity of COVID-19

It is documented that COVID-19 is a highly infectious virus. The basic reproduction rate (*R*_0_) was estimated early in the outbreak and found to range between 2.2 and 3.58. Several factors might contribute to its infectivity, one of which is the structure of the spike which contains receptor-binding domain (RBD). COVID-19 relies on the RBD in the spike to bind to a corresponding receptor in the host [39]. When the spike protein sequence of multiple CoVs was compared and aligned with the human angiotensin-converting enzyme-2 (ACE-2), it revealed the highest compatibility with COVID-19 [40]. The high affinity of COVID-19 RBD to human ACE-2 might also indicate the involvement of more intermediate hosts that culminate in mutations and better adaptation [40].

The second key factor for the COVID-19 infectivity is the mode of transmission. Human to human transmission was confirmed from the first infected contact cases found in Wuhan, China. It infects the respiratory system via the aerosol route [41]. However, the virus was later isolated from the stool of some patients, which ultimately indicates other routes of transmission such as water-borne and direct contact [42]. The novel virus sustains survival for a longer duration on multiple surfaces. It can also withstand survival on wood for 4 days, on steel, metal, glass, and paper for 5 days, and on plastic for 9 days according to a systematic review of several studies [43].

The mean incubation period of COVID-19 was estimated to be 5.2 days, with a range of 4.1–7.0 based on the first 10 cases in China, Hence, the quarantine period for COVID-19 was adopted to 14 days [44]. However, subsequent cases emerged with a longer incubation period. A study done on 50 patients in Wuhan reported prolonged incubation time in comparison to SARS-CoV and MERS it can be up to 24 days in the case of SARS-CoV2 [45]. A study on 1099 patients in 552 hospitals in China reported asymptomatic carriers with positive tests of the virus were reported among the patients’ contacts [46].

### Diagnostic techniques

In the case of coronavirus infections, as it is highly contagious, the diagnostic measures must be specific and should not rely on clinical manifestation only. In those cases, where symptoms are atypical, auxiliary examinations are needed for the proper diagnosis. Clinical diagnosis of coronavirus infections is mainly based on epidemiological history, clinical manifestation, and laboratory investigation.

### Epidemiological history

COVID-19 rapidly spreads across the major nations of the world. People are suspected to be COVID-positive based on their travel history and their contact with infected patients (particularly in the last 14 days). This is done because this virus can spread either through contact with certain bodily fluids, or can enter the body through the mouth, nose, or eyes when the suspected patient comes in contact with anything that has been in contact with an infected person. Rapid collection of data relating to these epidemiological factors can help in ascertaining whether the person needs to undergo the test or not.

### Clinical manifestation

The coronavirus infection shows initial symptoms which resemble those of common flu infection and include many symptoms which overlap with other common illnesses.

#### SARS

In the case of SARS, fever, cough, myalgia, malaise, and chills are observed as initial symptoms. However, in later stages, shortness of breath, tachypnea, or pleurisy is also found, but the sore throat was found to be less common. Watery diarrhea occurs in some patients in later stages [47].

#### MERS

In the case of MERS, a person might display symptoms after 5–6 days of exposure, but in some cases, the onset of symptoms might take 14 days. Symptoms of MERS include fever, cough, and shortness of breath. Few people also exhibit diarrhea, nausea, and vomiting. Severe complications like kidney failure and pneumonia were observed in later stages [48].

#### SARS-CoV-2

Like any other coronavirus infection, early symptoms of COVID-19 are nonspecific and easily relatable to mild illness observed during the common flu. In general, it affects the respiratory system and causes dry cough, fever, dyspnea, sore throat, runny nose, chest pain, sputum, headache, nausea, vomiting, diarrhea, abdominal pain, and fatigue. Fever was observed as onset symptom in most (~ 97%) of the patients; ~ 93% of patients suffered dry cough, ~ 90% experienced dyspnea, ~ 75% had a sore throat, whereas a small percentage showed signs like runny nose, and fatigue (~ 11–44%) [48]. Although dyspnea is experienced by many patients, a study has revealed that out of 50 suspected patients, only 15 are found to be COVID positive, though none of them had severe dyspnea [49]. In the current situation, it is of utmost importance to fully elucidate the range of disease—from asymptomatic to severe and to understand the risk factors associated with the progression of the disease [50].

### Diagnostic techniques for coronaviruses

Polymerase chain reaction (PCR), with high sensitivity and specificity, is considered a gold standard test for the molecular diagnosis of viral infections. In the case of Zika infections, there are no specific tests that are easily available. In most cases, diagnosis is based on clinical symptoms and epidemiological circumstances. However, after 3–5 days of the onset of symptoms, the body fluids of the infected person like blood, saliva, urine are collected for PCR, nucleic acid detection by reverse transcriptase-polymerase chain reaction (RT-PCR) (targeting the non-structural protein 5 genomic regions) [51]. Viral cultures are no longer recommended for screening [52].

Serological tests like ELISA and Plaque Reduction Neutralization Assay are also available [51]. Ebola fever is diagnosed using blood tests to detect the virus in the patient’s blood [53]. For MERS, PCR was also used. Throat swab, sputum, tracheal aspirate, or broncho-alveolar specimens are taken and stored at 28 °C and transported within 72 h to the reference laboratories, where they are subjected to real-time reverse-transcriptase–polymerase-chain-reaction (rRT-PCR) assays [48, 54, 55]. Even for SARS, at least two different clinical specimens, for example, nasopharyngeal and stool are collected to perform RT-PCR [47]. For all these tests, the results of RT-PCR tests are confirmed by measuring cycle-threshold values for viral load. Until now, genomics screens of animal populations have primarily been used to identify novel viruses in epidemiological situations.

## COVID-19 diagnostic techniques

### Nucleic acid testing

These tests detect specific nucleic acid sequences and are often used to detect viral infections. They detect genetic material that allows early diagnosis of disease as compared to detection of antigen or antibody because these immunological materials need a certain time to appear in the bloodstream. As the amount of genetic material to be detected is very low, there is always a need to amplify them before detection. However, a major drawback with these tests is that they detect the presence of viral RNA, and not the viable virus.

#### Real time-polymerase chain reaction (RT-PCR)

Similar to the diagnosis of other coronaviruses, rRT-PCR is used in the diagnosis of COVID-19 as well. Samples collected are generally throat swab, sputum, tracheal aspirate, or bronchoalveolar lavage specimens. However, serum is not accepted as a PCR sample [52]. For SARS-CoV-2, two technologies: high-throughput sequencing and rRT-PCR are used for the detection of nucleic acids of the pathogenic virus [56]. However, these techniques are not devoid of shortcomings—the use of high-throughput sequencing is dependent on advanced equipment, instrumentation, technical skills, and hence, amount to a very high price. Thus, rRT-PCR is used commonly and is considered to be an effective and straightforward method to analyze respiratory secretions and blood samples of COVID-19 patients for detection of the virus.

Real-time fluorescent RT-PCR is also used to detect nucleic acid of a novel coronavirus in respiratory secretions or blood samples [52, 57]. In PCR, genetic material extracted from these samples is amplified. Once the genetic material is obtained in sufficient quantity, conserved genetic codes of coronavirus are detected. Probes for detection are based on the initial gene sequence released. These specific probe sequences [in ORF1 (human RNA polymerase protein), E gene (Envelope protein), and N gene (Nucleocapsid protein) regions] were released by CDC (Centers for Disease Control and Prevention) and recommended for use to carry out detection of SARS-CoV-2 [56, 58]. It was found that these primer and probe sequences have a perfect match with other SARS-CoV-2 genome sequences available from Global Initiative on Sharing All Influenza Data (GISAID). Table 2 demonstrates different primers and probes that are used in RT-PCR. Many of the commercial PCR kits contains three assays. Each assay targets a different gene in the virus, so if the virus does mutate, the chances of all three genes mutating together are low. Thus, if one or two of these assays are positive, the results are recorded to be inconclusive. The SARS-CoV genomic RNA is used as a positive control. Positive confirmatory results are derived if both targets are positive. If positive results are found, it is suggested to repeat the test. A one-step TaqMan-based fluorescence signal (RT-qPCR) assay to detect both the regions (ORF1 and N gene) of the viral genome separately was also described [57]. In another study, a RT-qPCR (non-probes SYBR based fluorescence signal) gave positive results of SARS-CoV-2 at a high rate with saliva samples that were self-collected by the patients which suggests that saliva can be a promising non-invasive specimen for the SARS-CoV-2 diagnosis [56].

**Table 2:** Primers and probes sequences used in RT-PCR [59]

Assay	Gene sequence
ORF1a (413 bp)	5′-TTCGGATGCTCGAACTGCACC-3′ (Sense)
	5′-CTTTACCAGCACGTGCTAGAAGG-3′ (Antisense)
ORF1b (132 bp)	5′-TGGGGYTTTACRGGTAACCT-3′ (Forward, Y = C/T; R = A/G)
	5′-AACRCGCTTAACAAAGCACTC-3′ (Reverse, R = A/G)
	5′-TAGTTGTGATGCWATCATGACTAG-3′ (W = A/T; 5′-FAM/ZEN/3′-IBHQ)
N gene (110 bp)	5′-TAATCAGACAAGGAACTGATTA-3′ (Forward)
	5′-CGAAGGTGTGACTTCCATG-3′ (Reverse)
	5′-GCAAATTGTGCAATTTGCGG-3′ (5′-FAM/ZEN/3′-IBHQ)

The reliability of PCR is still questionable as the quality of available SARS-CoV-2-nucleic acid detection kits differs greatly, and this test has a few shortcomings, as mentioned below:

- Due to its low detection rate for SARS-CoV-2, the test needs to be repeated at least 2 to 3 times in many cases. When the viral load is low, the detection rate is also low, leading to false-negative results. Patients with negative results were thus, confirmed to be infected after repeated swab tests (RT-qPCR). The study found that detection of SARS-CoV using RT-qPCR can only achieve a sensitivity of 50–79% [56].
- The reliability of test results depends on several clinical specimens collected, as one sample may not provide accurate outcomes.
- Test sensitivity depends on the protocol used. Variations in results are observed with the change in protocol.
- It is found that the sensitivity of the test is also based on the type of sample. In few cases, pharyngeal swabs produced negative results but bronchoalveolar lavage samples from the same patients gave positive results [52]. One study suggests that the time of collecting sample might also be of critical importance, as clinical sensitivity of RT-PCR was 100% on swabs taken on days 1–5 of symptoms, with no difference when compared with the swab and sputum samples taken simultaneously [47, 60].
- There are certain biological safety hazards accompanied by the retention and operation of patient samples.
- Nucleic acid detection steps after nucleic acid amplification are too clumsy and burdensome.
- Long waiting time for results. It takes 1 day or longer to obtain the results after sampling.
- The cost of the testing platforms is also a drawback.
- It requires specialized laboratory equipment and skilled technicians.
- Only a positive or negative diagnosis can be made, but the severity of the disease and its progression cannot be judged.
- Contaminated reagents give a false-positive test.
- Cannot detect resolved infection, i.e. if a person has had the infection and cleared the virus, this condition cannot be detected by PCR, as PCR only detects the presence of the active virus.
- According to a report, few cases tested positive after 2 successive negative results. It is not yet fully understood if this is due to reactivation or reinfection or just because of a testing error [61].
- As of now in the outbreak situation, the supply of the reagents is far too low as compared to the demand. The testing capacity of health care centers is very low to meet the requirement of people, waiting for PCR to detect SARS-CoV-2. Due to this shortcoming, many patients have not been diagnosed promptly and thus, have missed the chance of early isolation and early treatment.
- Due to the need for rapid and precise unerring SARS-CoV-2 testing, scientists are trying to bring in improved and more specific PCR tests for COVID-19; e.g. researchers at the University of Innsbruck (Austria), working in collaboration with Sinsoma GmbH (Völs, Austria), have developed a PCR test for SARS-CoV-2 detection, where they have combined endpoint PCR with capillary electrophoresis [62].

#### Loop-mediated isothermal amplification (LAMP)

LAMP differs from RT-PCR, as viral DNA copies are produced at a constant temperature of 60–65 °C, instead of using a series of temperature changes, and its results can be seen visually without the use of the machine. This test is rapid and produces results within 2–3 h. The amount of DNA produced in LAMP is much higher than in RT-PCR. Compared to RT-PCR, LAMP is a newer technique, is technically simple, and can be done within hospital laboratories that make it a more potential technique for COVID-19 detection. At this stage there is not much data available about its practical use as there are ongoing clinical trials to support the test. LAMP can detect current infections of disease, allowing medical staff to determine currently infected individuals [63].

There are few limitations associated with LAMP like:

- The principle behind building these tests is more difficult than RT-PCR.
- Cannot detect resolved infection, as it relies on capturing and detection of the virus. Thus, there is a possibility of missing patients who have recovered.
- Multiple samples are needed as the density of viral distribution differs across the respiratory tract, so even if a person is infected, the virus may only be detectable in sputum or nasopharyngeal swab but not necessarily at both locations at the same time.
- LAMP tests for COVID-19 can only tell if a person is currently infected with this particular coronavirus.
- It cannot provide information on other diseases or symptoms and does not reveal if a patient has been previously infected with the virus or if a patient has any immunity against the virus.

### CRISPR-based detection of COVID-19

Limitations of current diagnostics are turning researchers towards the use of newer techniques like Clustered Regularly Interspaced Palindromic Repeats (CRISPR). CRISPR consists of two components—Cas enzymes (CRISPR associated enzyme) (that cleaves specific strands of nucleic acid that are complementary to the CRISPR sequence) and a guide RNA (that recognizes the required sequence). CRISPR-Cas is a tool for cutting DNA at a specifically targeted location. The targeting ability of guide RNA (gRNA) is used in CRISPR diagnostics. In CRISPR diagnostics for COVID-19, CRISPR-Cas components are modified, for example, by attaching a fluorescent protein to the complex to emit a fluorescent signal in response to positive or negative detection of the target genetic sequence.

#### CRISPR based DETECTR assay

Recently a technology DNA Endonuclease Targeted CRISPR Trans Reporter (DETECTR) is being adapted to detect SARS-CoV-2 [64]. In this technique, RNA extracted from nasopharyngeal or oropharyngeal swabs undergoes simultaneous reverse transcription and isothermal amplification (using RT-LAMP), where viral N gene or E gene is amplified, followed by Cas12 detection of predefined coronavirus sequences. Cas12a-gRNA complex is designed to detect N gene or E gene [65]. Cas12a-gRNA complex binds to the target sequence due to which Cas12a is activated and it starts cleaving reporter molecule i.e. fluorescently labeled ssDNA. Later, fluorescence is visually detected. Different approaches are used for visual detection like later-flow strips, agarose gel detection, and fluorescence visualization.

#### CRISPR based SHERLOCK test

After SARS-CoV-2 genome characterization, Specific High-sensitivity Enzymatic Reporter unlocking (SHERLOCK) is under revamp to detect COVID-19 [65]. Extracted RNA is subjected to Isothermal Recombinase Polymerase Amplification (RPA), where it amplifies viral Orf1ab or S gene. In this test, gRNA is designed to detect the virus Orf1ab gene or S gene. Cas13a uses complementary crRNA (CRISPR RNA) to bind to the target sequence. Once this binding occurs, it activates the Cas13a enzyme which degrades the nearby RNA and the fluorescent RNA molecules (which are included to generate detectable signal), resulting in fluorescence. It is further incorporated with different detection approaches like lateral-flow read-out strips. Illustrates CRISPR-based DETECTR and SHERLOCK techniques.

#### Other CRISPR techniques

CRISPR diagnosis is very precise in targeting, highly specific, visual, faster, user-friendly, and low-cost alternative to PCR. However, its detection sensitivity is lower as compared to the qRT-PCR. To overcome limitations associated with it, researchers are continuously trying to improve this technique by using different strategies. AIOD-CRISPR (All-in-one dual CRISPR/Cas12a) uses two gRNAs for improved specificity; it is rapid and ultrasensitive [66]. FELUDA (FnCas9 Editor Linked Uniform Detection Assay) is a rapid, field-deployable nucleobase detection and identification technique using FnCas9. It is highly sensitive to the presence and position of mismatches within DNA [62]. CREST (Cas13-based, Rugged, Equitable, Scalable Testing) is a scalable cost-effective, easy-to-deploy, Cas13a based technique combining the quality of PCR with CRISPR-based detection [67].

However, these nucleic acid detection tests cannot be considered as confirmatory tests because patients with negative results of nucleic acid detection for SARS-CoV-2 may present positive chest CT findings. Thus, a clinically suspected patient with negative nucleic acid detection but positive imaging results should be isolated and treated as soon as possible.

### Chest radiographs

#### Computed tomography (CT)

Medical professionals are proposing CT scans as an obligatory auxiliary diagnostic method, as they are found to be more sensitive. In comparison to PCR, CT scans are more reliable as they are rapid, less time-consuming and till now, have shown a high positive rate. In high-frequency infected areas. Clinicians suggest that CT scans have appreciable value for COVID-19 diagnosis. When RT-PCR yields negative results for suspected individuals, showing COVID-19 symptoms, diagnosis with a combination of CT scan and repeated RT-PCR would be far more beneficial. Particularly, the high-resolution chest CT is important for early diagnosis and evaluation of disease progression of COVID-19 patients, as this disease has different imaging manifestations at different stages, which are mainly related to pathogenesis. Very few cases have negative CT findings at the early stage. Several studies have analyzed chest CT images of patients infected with SARS-CoV-2; at the initial stage, viral pneumonia is subject to affect the terminal bronchioles and pulmonary parenchyma surrounding them. Subsequently, it reaches infiltration of pulmonary lobules and as it progresses towards the advanced stage, the alveoli are severely damaged.

Typical CT images show different imaging at different stages; therefore, it is more helpful in tracking the progression of the disease. Some of the imaging results for COVID-19 are described below and summarized in Table 3.

- The lesions at the early stage of COVID-19 are relatively localized and mainly manifest as inflammatory infiltration, restricted to the sub-pleural or peribroncho-vascular regions of one lung or both lungs, exhibiting patchy or segmental pure ground-glass opacity (GGOs) with vascular dilation and segmental/patchy bilateral pulmonary parenchymal ground-glass opacity (86–93%) [49, 56].
- CT images in the later stage showed an increased stretch of pure GGOs, few consolidated regions and GGOs around these lesions (a distinct feature of progression), and involvement of multiple lobes, with consolidative pulmonary opacities (nearly 65%). Single or multiple lesions are also observed; vascular enlargement in the lesion (71.3%) with peripheral distribution (87.1%) and bilateral involvement (82.2%) are also visualized. Lesions are found to be predominant in the lower lung (54.5%) and are multifocal. Peri-bronchovascular and sub-pleural distribution of reticular marking was observed along with crazy-paving pattern and interlobular septal thickening. In rare cases, mediastinal lymph nodes and pleural effusion were observed.
- CT imaging at the advanced stage of COVID-19 was found to be similar to other types of pneumonia. The aspect of CT images at this stage is called lung whiteout, which shows the presence of diffused lesions in both lungs—Lesions are generally consolidated, and GGOs were found surrounding consolidated lesions, which are mostly accompanied by parenchymal bands and occasionally, by a small amount of pleural effusion.

**Table 3:** CT image observations at different stages of the COVID-19 [49, 68]

Early-stage	Progressive stage	Advance stage
Localized lesions are mostly restricted to pleural or peribroncho-vascular regions of one lung or both lungs Patchy pure GGOs Focal GGOs Vascular dilation	Consolidation Stretch of pure GGOs Bilateral peripheral GGOs Vascular enlargement in lesions The predominant lesion in the lower lung Interlobular septal thickening, with crazy-paving patterns Reticular marking in sub-pleural or peribroncho-vascular regions Rare pleural effusion	Diffused lesions in both lungs and expansion of bilateral pulmonary lesions Dense and enlarged consolidation GGOs surrounding consolidation Parenchymal bands Pleural effusion

But we cannot completely rely on CT imaging because in some cases, it is difficult to differentiate COVID-19 from other illnesses such as SARS, MERS, cytomegalovirus infection, influenza, adenovirus infection, and other viral and bacterial pneumonia by mere visual examination, as they can have same CT image. CT scans also have some shortcomings, such as the hysteresis of abnormal CT imaging. Therefore, clinical manifestations, contact history, and laboratory tests must be taken into consideration together to make the final diagnosis.

#### Chest X-ray

In the case of chest X-ray, poster anterior and lateral views are observed for architectural distortion, traction bronchiectasis, and pleural effusions, which may reflect the viral load and virulence of COVID-19 [48]. Chest X-rays are helpful to conclude viral load to some extent. Viral load and virulence were found to be statistically different between the emergency group and non-emergency group; therefore, this type of X-ray will be very helpful to identify the emergency type disease. Hence, the viral load could be taken into consideration to identify the severity of COVID-19 pneumonia.

### Serological tests

Serological tests use blood samples and the immune response of the patients to identify whether the person has been exposed to a particular pathogen. Rigorous research is going on to make these tests practically available for COVID-19 detection. Unlike nucleic acid detection, these tests will indicate if a person had an infection at some point and had subsequently recovered from it. If improved in the context of COVID-19, these tests will help study the prevalence of the pandemic in any population and assessing ‘herd’ immunity, which will further help to decide measures of social distancing and quarantine. Even though these tests are rapid and easy, their use is limited for SARS-CoV-2 detection, because a person’s immune response takes time to mount a detectable antibody response. Serological tests will be a useful tool to elucidate the link between cases.

Antibodies are usually produced against the most abundant protein, that acts as the antigen of the virus. Tests that detect antibodies against this protein would be more sensitive; thus, knowledge of the crucial viral proteins is important (e.g. viral coat protein). But there is a possibility of the antibodies cross-reacting with another coronavirus. Therefore, tests that detect antibodies to specific proteins like host-attachment protein RBD-S (Receptor-Binding Domain of S) would be more specific. Hence, the use of both antigens (RBD-S and viral coat protein) will result in a much robust test [69]. SARS-CoV-2 spike protein possesses few unique regions and is thus a potential antigen for the development of COVID-19 diagnostics [70].

Detecting viral protein (Ag) is a new approach, where monoclonal antibodies specific for viral protein are used and results can be visualized using chromatography; however, these tests require a high viral count to generate a proper result. For antigen and antibody detection tests, it is necessary to study those proteins that are crucial for the virus. So that they can be either used to develop monoclonal antibodies against them (to be used in antigen detection test), or these proteins can themselves be used in antibody detection tests. The most challenging part here is expressing these crucial proteins in the actual correct form. The following techniques are used in serological tests:

#### Lateral flow assay

Lateral flow assay (also known as immunochromatography) is used for the detection of proteins like antibodies, viral antigens, and small molecules. It involves the movement of antigens/Ag–Ab complex/antibodies through a support medium such as micro-structured polymer, nitrocellulose paper, filter paper, or agarose. Researchers are working to improve these tests in response to the rising diagnostic demand of COVID-19 in the current pandemic. In the case of COVID-19, these tests are designed to detect antibodies (IgM and IgG) and viral antigens. A drop of blood (finger prick) or saliva or nasal fluid is collected as a sample. These tests detect the infection by observing the patient’s antibody response to the virus, but it has a drawback that it cannot distinguish between current and any previous infection. Lateral flow tests are rapid, small, portable, easy-to-use tests with no requirement of skilled personnel and advanced laboratories [71].

Lateral flow for antigen is an advanced approach; successful commercial launch of these tests will be a great achievement to contain this pandemic as it will detect virus directly from a single sample without any amplification and thus, consuming less time. A Canada-based biotechnology company “Sona Nanotech Inc.” claims to have developed a lateral flow test using nanoparticles, to identify the SARS-CoV-2 within 15 min. They are trying to develop this quick-response lateral flow test for screening of COVID-19 patients. To beat the high-cost issues, this test is expected to cost ~ $50. Sona Nanotech will incorporate its exclusive nanorod technology into a disposable lateral flow test platform and this test can be handled by any unskilled person with no need for any lab equipment. It will be a huge success in the screening of triage individuals [72].

Despite all these advantages, this high-end technique is not devoid of drawbacks and has few disadvantages associated, such as:

- As the disease is new, not much data is available about its accuracy for the detection of SARS-CoV-2.
- Further tests are needed to find out whether the infection is current or previous.
- It is expensive
- Time-consuming for large batches.

#### Enzyme-linked immunosorbent assay (ELISA)

ELISA is a lab-based biochemical technique commonly used to detect antigens or antibodies. It has high throughput as it is done in a batch of 96 assays. This is a major advantage in the context of the current pandemic, COVID-19, where a large number of samples need to be tested in less time. Whole blood, plasma, or serum from a patient is collected as a sample in which antibodies (IgM and IgG) produced against SARS-CoV-2 are detected. Ninety-six wells on a plate are coated with viral protein of interest (e.g. Spike protein) and allowed to cross-react with the collected samples; if the sample contains antibodies to the viral protein, they will bind together. Later on, enzyme-labeled secondary antibodies are added which will subsequently bind to Ag-Ab complex and give color reaction or fluorescent-based readout (based on the label tagged with the secondary antibody). Besides, for diagnosing COVID-19, ELISA provides important information for viral infection control, which is the evaluation of the number of people infected in a population. ELISA-based IgM and IgG antibody tests have ≥ 95% specificity for the diagnosis of COVID-19. Commercial use of ELISA for COVID-19 includes a dual ELISA test that detects specific IgA and IgG against the virus in the blood of infected patients [71].

ELISA is a simple, quick (1–3 h) and cheap test, with the feasibility of testing multiple samples at the same time. Although it is well documented and widely used method by researchers of various fields, it is not yet established for SARS-CoV-2 testing. But the bright side is that many companies are working hard to validate them commercially.

#### Chemiluminescent immunoassay (CLIA)

CLIA is also under trial to make it available for COVID-19 diagnosis. This is a quantitative test having similarities in principle with ELISA. In this test, enzyme-labeled secondary antibodies are used that allow light-based, luminescent read-out. Modified versions of this test are being tried using magnetic or protein-coated micro particles, for example, a peptide-based luminescent immunoassay to detect IgG and IgM. It is suggested that this test in combination with PCR will improve the accuracy of the COVID-19 diagnosis manifold [73].

#### Neutralization assay

In neutralization assay, the patient’s antibodies are tested for effectiveness against SARS-CoV-2. Whole blood, plasma, or serum from the patient is collected and the presence of neutralizing antibodies (NAbs) is checked. NAbs play an important role in viral clearance as they can block viral infection. Cells that allow SARS-CoV-2 to grow are cultured, grown with decreasing concentration of patient’s sample (antibodies), and visualized to check how many antibodies can block viral growth [74]. Neutralization assay is also necessary to rule out antibodies cross-reacting with another coronavirus. Limited literature is available about NAbs in COVID-19 patients. As transfusion of convalescent plasma/serum from recovered patients is an outlook, this assay is currently being considered as an auspicious therapy in many countries and is very useful to determine whether the antibodies in the convalescent plasma are effective or not. It is indeed praiseworthy that some researchers have successfully developed specifically sensitive plaque reduction neutralization assay in such a short time, and suggested that a simple micro-neutralization assay has enough sensitivity for population study [60].

#### Nano-sensors

Currently, at public places, thermal screening guns are used to screen out people with a high fever. This can be replaced by nano-sensor diagnostic tools that will use nano-sensor technology to detect nucleocapsid protein specific for SARS-CoV-2 and will give specific results for this virus, instead of generalized thermal screening. It is still under the research stage, but if developed successfully, it will produce a result within a very short period of time [75].

#### Blood test

Laboratory findings have suggested certain abnormal counts in blood cells and enzymes in COVID-19 infection that include lymphopenia (70%), prolonged prothrombin time (58%), increased values of lactate dehydrogenase, liver enzymes and muscle enzymes, and decreased or normal white cell count or decreased lymphocyte count in the early period of infection [6]. These type of blood tests can also be used to as a diagnostic tool for the detection of COVID-19.

#### Viral sequencing

WHO has strongly recommended that along with the confirmed presence of a virus, regular sequencing must be done to monitor possible mutations that might affect different diagnostic tests [76]. With the emergence of deadlier variants, viral sequencing is an important step to fasten our pace in fighting the COVID-19 pandemic.

### Diagnostic prospective

Given the shortcomings of the currently available diagnosis for COVID-19, there is indeed a great need for the development of in vitro diagnostic platforms, capable of accurate, rapid, and field-friendly detection. There is a need to develop and validate sensitive and specific auxiliary tests using different diagnostic methods such as ELISA, Lateral Flow assay, improved Molecular diagnostics (CRISPR), Colorimetric tests, Chemiluminescence Immunoassays, and neutralization assays.

For improved diagnostics better understanding of the pathogenesis, infectivity, and life cycle of the virus and the disease is required. In the context of diagnosis, various studies like finding a relation between viral concentration and disease severity, developing useful serological assays, and comparative study of molecular and serological assays have to be considered.

One study has performed a recombinant immunofluorescence assay to deduce the specific reactivity against recombinant spike protein of SARS-CoV-2 [60]. However, there is still an urgent need to develop serological tests that can estimate the current infections in general populations. For suspected cases, rapid antigen detection, and other investigations should be adopted for evaluating common respiratory pathogens and non-infectious conditions. Serum antibody tests should be done in asymptomatic high-risk individuals with a history of exposure to patients with COVID-19 pneumonia to facilitate early identification of the disease. Additional tests, such as complete blood cell count and routine microbiology, including molecular testing for other respiratory viruses, can be handled using universal precautions in hospital laboratories. Immunological detection tests that target viral antigens or antibodies against them must be used in laboratories a soon as possible.

## Conclusions

The novel coronavirus (SARS-CoV-2) which emerged in China in December 2019 has been classified as the seventh beta coronavirus. It manifested in the early cases as acute respiratory distress syndrome. Later, it appeared with different presentations ranging from asymptomatic individuals, mild flu to the most severe presentation of acute respiratory distress illness and ultimately respiratory failure. The high infectivity of this novel virus might be due to the high compatibility of its spike to the binding site (ACE2 receptors) in human pneumocytes, the long survival duration on inanimate surfaces, the long incubation period, and the documented human-to-human transmission. Techniques like lateral flow assay, improved molecular diagnostics (CRISPR), colorimetric tests, chemiluminescence immunoassays, neutralization assays, RT-PCR, and chest CT scan are the major diagnostic techniques for COVID-19, but all the techniques have some limitations. Relying only on one or two techniques affects the early diagnosis and isolation of the infected persons. The undetected infectious persons pose a major threat in managing, controlling, and curbing the disease outbreak as evident from the resurging of COVID-19 cases in certain regions of the world. The present gloomy scenario mandates clinical manifestations, contact tracing, and laboratory tests as important parameters that need to be taken into consideration to make the final diagnosis.

## Data Availability

All data and materials are available on request to the corresponding author.

## Abbreviations

RNA: Ribonucleic acid
SARS: Severe acute respiratory syndrome
MERS: Middle East respiratory syndrome
COVID-19: Coronavirus disease 2019
EBOLA: *Ebola* Virus disease
ZIKA: Zika virus disease
CDC: Centers for Disease Control and Prevention
NCEZID: National Center for Emerging and Zoonotic Infectious Disease
WHO: World Health Organization
RTC: Replication transcription complex
TRS: Transcription regulatory sequences
ORF: Open reading frame
RBD: Receptor binding domain
ACE: Angiotensin converting enzyme
PCR: Polymerase chain reaction
RT-PCR: Reverse transcriptase-polymerase chain reaction
rRT-PCR: Real time reverse transcriptase-polymerase chain reaction
GISAID: Global Initiative on Sharing All Influenza Data
GGO: Ground-glass opacity
ELISA: Enzyme-linked immunosorbent assay
CLIA: Chemiluminescent immunoassay
FELUDA: FnCas9 editor linked uniform detection assay
CREST: Cas13-based, Rugged, Equitable, Scalable Testing
SHERLOCK: Specific High-sensitivity Enzymatic Reporter unlocking
CRISPR: Clustered Regularly Interspaced Palindromic Repeats
